# A comparison of missing data procedures for addressing selection bias in HIV sentinel surveillance data

**DOI:** 10.1186/1478-7954-11-12

**Published:** 2013-07-24

**Authors:** Marie Ng, Emmanuela Gakidou, Christopher JL Murray, Stephen S Lim

**Affiliations:** 1 Institute for Health Metrics and Evaluation, University of Washington, Seattle, USA

**Keywords:** Selection bias, Simulations, Missing data, Multiple imputation, Complete-case analysis

## Abstract

**Background:**

Selection bias is common in clinic-based HIV surveillance. Clinics located in HIV hotspots are often the first to be chosen and monitored, while clinics in less prevalent areas are added to the surveillance system later on. Consequently, the estimated HIV prevalence based on clinic data is substantially distorted, with markedly higher HIV prevalence in the earlier periods and trends that reveal much more dramatic declines than actually occur.

**Methods:**

Using simulations, we compare and contrast the performance of the various approaches and models for handling selection bias in clinic-based HIV surveillance. In particular, we compare the application of complete-case analysis and multiple imputation (MI). Several models are considered for each of the approaches. We demonstrate the application of the methods through sentinel surveillance data collected between 2002 and 2008 from India.

**Results:**

Simulations suggested that selection bias, if not handled properly, can lead to biased estimates of HIV prevalence trends and inaccurate evaluation of program impact. Complete-case analysis and MI differed considerably in their ability to handle selection bias. In scenarios where HIV prevalence remained constant over time (i.e. *β* = 0), the estimated ${\hat{\beta }}_{1}$ derived from MI tended to be biased downward. Depending on the imputation model used, the estimated bias ranged from −1.883 to −0.048 in logit prevalence. Furthermore, as the level of selection bias intensified, the extent of bias also increased. In contrast, the estimates yielded by complete-case analysis were relatively unbiased and stable across the various scenarios. The estimated bias ranged from −0.002 to 0.002 in logit prevalence.

**Conclusions:**

Given that selection bias is common in clinic-based HIV surveillance, when analyzing data from such sources appropriate adjustment methods need to be applied. The results in this paper suggest that indiscriminant application of imputation models can lead to biased results.

## Background

In the last decade, there has been an exponential increase in development assistance for health targeted at HIV with a correspondingly massive scale-up in prevention and treatment programs to combat HIV worldwide [1,2]. While intervention programs have often been evaluated in terms of their impact on behavioral indicators such as condom use and utilization of services, it is critical to understand the impact of increased funding and the array of intervention programs on population health. This is best achieved by examining the relationship between program inputs and changes in HIV incidence or prevalence over time.

Given the difficulties in tracking HIV incidence, in many low-and-middle-income countries, the extent of the HIV epidemic in the general population has primarily been monitored via clinic-based HIV surveillance programs that measure prevalence among women receiving antenatal care (ANC) [3]. Where available, these data are supplemented with population-based surveys of HIV prevalence, which often consist of more representative samples of the general population.

Previous studies have highlighted the problem of selection bias associated with ANC-based surveillance [4-6]; that is, the *level* of HIV prevalence from ANC sites has been shown to be markedly higher than corresponding population-based surveys, as women attending ANC are at higher risk of HIV than the general population. Recognition of this issue led to revisions by UNAIDS of trends in HIV prevalence in 2003, and corrections for this bias are included as part of the Epidemic Projection Package (EPP) [7]. Corrections for this bias revised the total number of people living with HIV worldwide down from the previously estimated 40 million to 35 million in 2001 [8].

In addition to the selection bias with respect to sampled women, another form of selection bias which is less recognized is the selection bias with regard to sites [9]. In many countries, ANC clinics located in HIV hotspots are often chosen and incorporated into surveillance systems earlier than clinics in locations where HIV is believed to be less prevalent [10-16]. In India, for example, national sentinel surveillance for HIV/AIDS began in 1992 and sites included in the surveillance system were located mainly in six high-prevalence states [17]. Over the years, the surveillance network has expanded to include the other 29 states believed to have lower prevalence (See Table 1).

**Table 1:** **Number of sentinel sites by year and type from 1998 to 2008, India**[1]

	**Year**										
**Site type/year**	**1998**	**1999**	**2000**	**2001**	**2002**	**2003**	**2004**	**2005**	**2006**	**2007**	**2008**
STD	76	75	98	133	166	163	171	175	251	248	217
ANC	92	93	111	172	200	266	268	267	470	484	498
IDU	5	6	10	10	13	18	24	30	51	52	61
MSM	-	-	3	3	3	9	15	18	31	40	67
FSW	1	1	2	2	2	32	42	83	138	137	194
ANC (Rural)	-	-	-	-	-	210	122	124	158	162	162
TB	2	2	-	-	-	-	7	4	-	-	-
Migrant	-	-	-	-	-	-	-	1	6	3	8
Eunuchs	-	-	-	-	-	-	-	1	1	1	1
Truckers	-	-	-	-	-	-	-	-	15	7	7
Fisher Folk	-	-	-	-	-	-	-	-	1	-	-
Others (Seamen)	-	-	-	-	-	1	-	-	-	-	-
Total	176	177	224	320	384	699	649	703	1122	1134	1215

Site selection bias raises two issues: (1) It obscures the estimation of both the *level* and the *trend* of the HIV prevalence. For clinic-based surveillance systems that have evolved in such a way, HIV prevalence estimates for the earlier time periods will often be biased upward and appear relatively high. In later years, HIV prevalence estimates will be more representative with the addition of sites from low-prevalence areas. This exaggerated the estimated declines in HIV prevalence. (2) The lack of complete data from low prevalence areas poses challenges to the evaluation of the impacts of HIV intervention programs. Locations with relatively high HIV prevalence often receive intervention programs, whereas locations with lower HIV prevalence do not. Missing surveillance data in the low prevalence areas hinders the estimation of HIV trends in nonintervention locations, which makes it difficult to compare trends between intervention and nonintervention locations.

Few studies have explored the issue of site selection bias in HIV sentinel surveillance. In this paper, we aim to demonstrate the impact of this issue on the estimation of HIV prevalence trends and the evaluation of program impact. Specifically, given that the priority in surveillance often coincides with the priority in which intervention is implemented, it is of interest to understand how this will affect the comparisons of trends between intervention and nonintervention sites. Using both simulated and actual data, we compare two general approaches for handling the problem of site selection bias, namely complete-case analysis and multiple imputation.

## Methods

### Data

#### Simulation data

To compare the various approaches systematically, we simulate a set of scenarios that mimic how clinics are actually added to a national HIV surveillance system. We simulate HIV prevalence data for *N* = 30 sites with *n* = 400 observations per site and a surveillance period of *T* = 10 years. Among the 30 sites, the 15 sites with higher HIV prevalence were also intervention sites, while the 15 sites with lower HIV prevalence were nonintervention sites.

Let *y*_*st*_ be a vector with *n*_*st*_ clinic observations for site *s* at time *t*. Each observation, *y*_*ist*_, takes value 0 if the case is HIV negative or 1 if the case is HIV positive. Data are generated from the following model:

$$y_{\mathrm{ist}}\sim \mathrm{Bernoulli}\left(\;p_{\mathrm{st}}\right)$$

*P*_*st*_ is defined as

$$\mathrm{logit}\left(p_{\mathrm{st}}\right)=a_{s}+\mathrm{bt}$$

$$a_{s}=\left\{\begin{array}{l} \alpha^{0}+\alpha_{s}^{0}\;\mathrm{for}\;\mathrm{non}-\mathrm{intervention}\;\mathrm{sites}\;\\ \alpha^{I}+\alpha_{s}^{I}\;\mathrm{for}\;\mathrm{intervention}\;\mathrm{sites} \end{array}\right)$$

*α*^0^, *α*^1^, and *b* are the fixed effects intercepts and slope respectively. $\alpha_{s}^{0}$ and $\alpha_{s}^{I}$ are the site-specific random intercepts, which are assumed to be identically and independent distributed with a standard normal distribution.

Given that in most situations, sites with higher HIV prevalence would be given interventions, we assume different fixed effect intercepts for the intervention and nonintervention sites. Specifically, we set *α*^0^ = –3 and *α*^1^ = –1. As for the HIV prevalence trend, we assume that both intervention and nonintervention sites experience the same temporal trend with *b* = 0. In other words, HIV prevalence is assumed to be constant over time. This scenario aims to demonstrate the specificity of each method; that is, under different level of selection bias, how well a method performs in terms of guarding against false alarm. On the other hand, we focus on the high selection bias situation and consider two additional scenarios which aim to examine the sensitivity of each method. In one scenario, the temporal trend of the nonintervention sites are set to be constant, *b* = 0, whereas a declining trend is observed for intervention sites, *b* = –0.025. In another scenario, both nonintervention and intervention sites have declining trends but at differential rates, specifically *b* = –0.025 for nonintervention sites and *b* = –0.05 for intervention sites. In both scenarios, the difference in *b* (i.e., the difference in the slopes of time trend) for intervention and nonintervention sites is −0.025.

**Figure 1:**
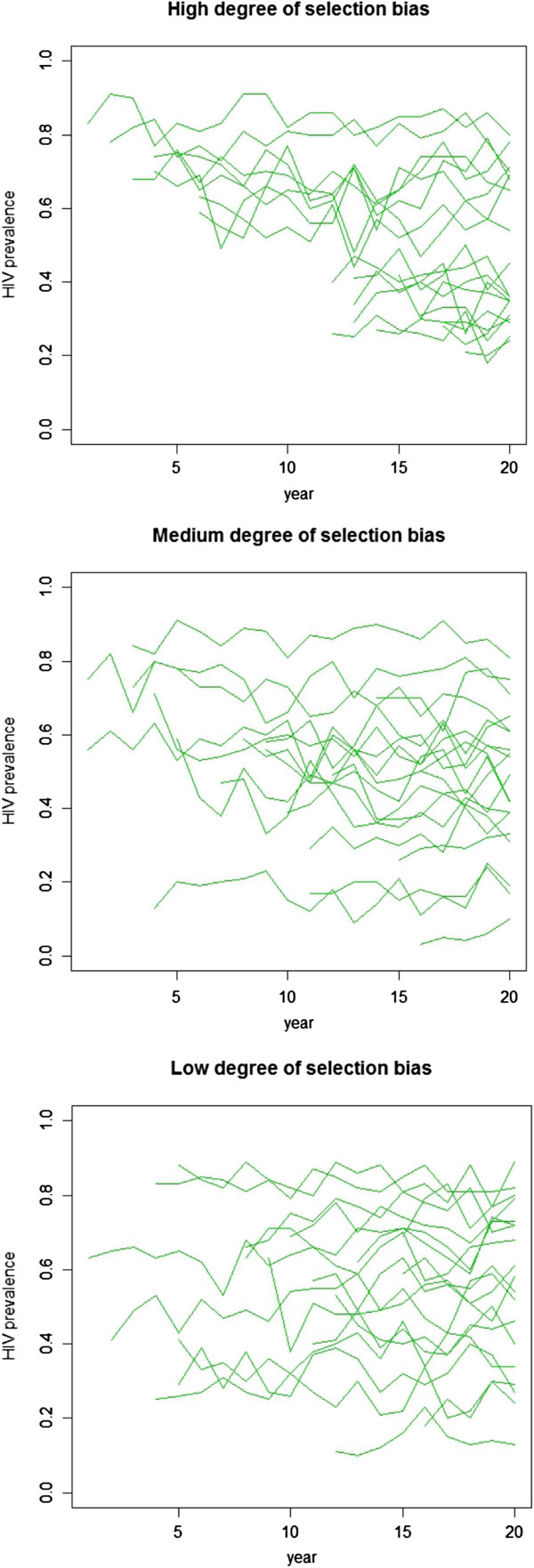
**Examples of simulated data with high, medium and low degree of selection bias.** Each line indicates the simulated HIV prevalence of a unique site.

Three levels of selection bias are simulated by varying the correlation between the initial prevalence level (i.e., prevalence at *t* = 0) and the year in which a site is selected and monitoring begins. In the high correlation cases (*ρ* = –0.95), sites with higher baseline prevalence levels are much more likely to be selected in the earlier periods. In the medium correlation cases (*ρ* = –0.5), there is a moderate association between the baseline prevalence level and the start year. In the low correlation cases (*ρ* = 0), sites are selected at random independent of their baseline prevalence level. Figure 1 shows an example of the simulated data with different degrees of selection bias. In all scenarios, approximately 70% of the sites are missing in the earlier period; by the middle of the time series, approximately 50% of the sites are observed. Toward the end of the time series, all sites are observed. Overall, the total proportion of missing data ranges from 45% to 55%. A total of 1,000 replications are performed.

#### India National AIDS Control Organization (NACO) sentinel surveillance data

**Figure 2:**
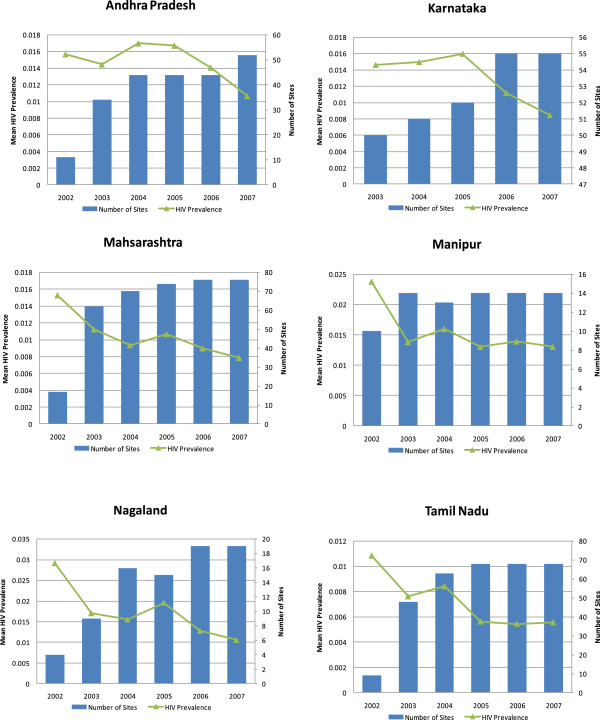
HIV prevalence trend and number of ANC sites in 6 Indian States.

In addition to the simulation data, we consider ANC sentinel surveillance data obtained from the National AIDS Control Organization (NACO) of India between 2002 and 2008. Figure 2 shows the HIV prevalence trends and the total number of ANC sites for the six states with the highest HIV prevalence. The six states are Andhra Pradesh, Karnataka, Maharashtra, Manipur, Nagaland, and Tamil Nadu. Together they accounted for approximately 64% of the HIV burden nationwide in 2006 [18]. As indicated by the histograms, relatively few sites were monitored in 2002. However, the number of sites grew continually over the five-year period. As the total numbers of sites increased, the HIV prevalence gradually declined in all six states.

The increase in the coverage of HIV surveillance also coincided with the implementation of an HIV prevention program. During the period of 2005 to 2008, a large-scale HIV prevention program known as the India AIDS Initiative (Avahan) was implemented in 80 of the 130 districts in the six high-prevalence states. Many of these districts were higher in HIV prevalence. We utilize this dataset to illustrate the application of the various approaches for handling missing data.

### Approaches and models

Two approaches are compared here: (1) complete-case analysis and (2) multiple imputation (MI). Complete-case analysis involves analyzing only observed data without imputation of missing data. On the other hand, MI involves replacing each missing datum by a set of *m* imputed values. Given a specific model for the response mechanism, values are imputed through random draws from the posterior predictive distribution. A unique strength of MI is that it captures not only sampling variability but also uncertainty in the imputation model. Furthermore, when applied properly, inferences based on MI possess desirable frequency properties such as high relative efficiency and desirable confidence coverage.

#### Imputation models

Here we consider three models for multiple imputation: (1) a fixed-effects model, (2) a mixed-effects model, and (3) a time series model. The ultimate goal is to identify a model that provides the most accurate estimates of HIV trends as well as intervention impact evaluation in the subsequent analysis.

#### Model 1: A generalized linear fixed-effects model

Again, Let ***y***_*st*_ be a vector with *n* clinic observations for site *s* at time *t*. Each observation, *y*_*ist*_, takes value 0 if the case is HIV negative or 1 if the case is HIV positive. ***y***_*st*_ is assumed to be fully observed for some site-years but missing for others. The observation can be modeled by

$$y_{\mathrm{ist}}\sim \mathrm{Bernoulli}\;\left(p_{\mathrm{st}}\right)$$

Here, parameter *p*_*st*_ is modeled by:

$$\mathrm{logit}\left(p_{\mathrm{st}}\right)=\beta_{0}+\beta_{1}t+\beta_{2}I+\beta_{3}I\times t$$

*I* is a dummy variable where it is 1 for intervention sites and 0 for nonintervention sites. This model assumes that the level and changes in HIV prevalence depend mainly on whether or not a site receives intervention. As mentioned earlier, missing data during the earlier period are related to the level of HIV prevalence, and the level of HIV prevalence is related to whether a site receives intervention. Therefore, intervention can be a useful predictor for the missing values.

#### Model 2: A generalized linear mixed-effects model

One limitation of Model 1 is that it may not adequately capture the potential heterogeneity of HIV prevalence across sites. Model 2 aims to overcome this by incorporating site-specific random effects. In particular, the parameter *p*_*st*_ is modeled by:

$$\mathrm{logit}\left(p_{\mathrm{st}}\right)=\beta_{0}+\beta_{1}t+\beta_{2}I+\beta_{3}I\times t+\alpha_{0s}+\alpha_{1s}t$$

*β*_*j*_ are fixed effect coefficients and $\alpha_{0s}$ and $\alpha_{1s}$ are the site-specific random intercept and slope. This model assumes not only that the level and trend of HIV prevalence differ between intervention and nonintervention sites, but also that they may vary across sites within each of these categories. Considering that HIV prevalence often varies substantially across sites, this model may yield more accurate imputed values.

#### Model 3: A lead-variable model

Let *C*_*st*_ be the total number of HIV positive observations and *n*_*st*_ be the total number of observations for site *s* at time *t.* The HIV prevalence for each site-year can be estimated by $p_{\mathrm{st}}=\frac{C_{\mathrm{st}}}{n_{\mathrm{st}}}$. An alternative way to present the data is through a two-way table with the sites as row (*s* = 1,⋯,*S*) and years as column (*t* = 1,⋯,*T*).

$$\begin{array}{ccc} p_{11} & \cdots & p_{1T}\\ p_{21} & \cdots & p_{2T}\\ \vdots & \cdots & \vdots \\ p_{S1} & \cdots & p_{\mathrm{ST}} \end{array}$$

*P*_*st*_ is observed for some site-years but missing for others. The problem of missing data resulting from site selection bias is most severe in earlier years of the surveillance period. As time progresses, the proportion of missingness is gradually reduced. This missing data pattern can be considered as monotone missingness. Model 3 takes advantage of this missing data pattern and imputes missing data in the earlier period based on complete data from later years. Specifically, we apply the following model to each column of the two-way table. Instead of a generalized linear model, we use a linear model. Beginning from the end of the surveillance period, use a lead value of *P*_*s,t+*1_ to predict the value of *P*_*st*_.

$$\mathrm{logit}\left(p_{\mathrm{st}}\right)=\beta_{0}+\beta_{1}\mathrm{logit}\left(p_{s,t+1}\right)+\varepsilon_{\mathrm{st}}$$

Based on the imputed value for the missing *P*_*st*_ , the missing *y*_*st*_ are imputed.

#### Analysis model

The output of multiple imputation is *m* complete datasets, which can be analyzed using complete-data methods. The following analysis model is fitted to each complete data set.

$$\mathrm{logit}\left(p_{\mathrm{st}}\right)=\beta_{0}+\beta_{1}t+\beta_{2}I+\beta_{3}I\times t+\alpha_{0s}+\alpha_{1s}t$$

For the purpose of this study, the key parameters of interest are *β*_1_ and *β*_3_. These parameters reflect the temporal changes in HIV prevalence and potential program impact. In particular for the simulated dataset, it is assumed that HIV prevalence remains the same over the years and does not differ between intervention and nonintervention sites. In other words, both *β*_1_ and *β*_3_ are expected to be close to zero. Any substantial deviation will be an indication of the adverse effect of site-selection bias. The coefficient estimates derived from the *m* complete data sets are combined using Rubin’s rule [19]:

The estimates for *β*_*j*_ and its standard error are obtained by:

$${\bar{\beta }}_{j}=\frac{1}{M}\overset{M}{\underset{m=1}{\sum }}\beta_{j}{}^{\left(m\right)}\;$$

$$\mathrm{SE}{\;}_{\beta_{j}}^{2}=\frac{1}{M}\overset{M}{\underset{m=1}{\sum }}\mathrm{SE}{\left(\beta_{j}{}^{\left(m\right)}\right)}^{2}+S_{{\hat{\beta }}_{m}}^{2}\left(1+\frac{1}{M}\;\right)\;$$

where M is the total number of imputed data sets, ${\hat{\beta }}_{j}{}^{\left(m\right)}$ is the estimate of *β*_*j*_ derived from the *m*^th^ imputed data set and $\;S_{{\hat{\beta }}_{m}}^{2}=\frac{1}{M}\overset{M}{\underset{m=1}{\sum }}{\left(\beta_{j}{}^{\left(m\right)}-{\bar{\beta }}_{j}\;\right)}^{2}\;$

The confidence interval for *β*_*j*_ is calculated using the normal approximation:

$${\bar{\beta }}_{j}\pm t_{\mathrm{crit}}SE_{\beta_{j}}\;$$

where *t*_*crit*_ is the 0.975 quantile of the *t*-distribution with degrees of freedom (d.f.) derived based on a Satterthwaite approximation [20]

$$\;d.f.=\left(M-1\right){\left\{1+{\left[\left(1+\frac{1}{M}\right)\frac{S_{{\hat{\beta }}_{m}}^{2}}{\frac{1}{M}\sum_{m=1}^{M}\mathrm{SE}{\left({\hat{\beta }}_{j}{}^{\left(m\right)}\right)}^{2}}\right]}^{-1}\right\}}^{2}\;$$

#### Complete-case analysis

For the complete-case analysis, in addition to applying the mixed-effects analysis model described above to the completely observed data, we consider a simpler model with only fixed effects:

$$\mathrm{logit}\left(p_{\mathrm{st}}\right)=\beta_{0}+\beta_{1}t+\beta_{2}I+\beta_{3}I\times t$$

The purpose of considering this alternative model is to explore the sensitivity of the approach model to the different specifications of the analysis model.

We should note that for the situation at hand, the unit of observation is site-year. Therefore, in the complete-case analysis, we retain data from all observed site-year. If datum is missing for a site in a specific year, we drop only the missing observation of that specific year for that site.

Simulations and the analysis of NACO data were performed with the R statistical program, version 2.15.1 (http://www.r-project.org).

## Results

### Simulation results

Considerable variation was found in the outcomes of the different approaches. Overall, complete-case analysis with the mixed-effects model yielded the most unbiased coefficient estimates and provided the best probability coverage of confidence intervals. Moreover, among the three models considered for MI, Model 1 performed best. For example, in situations where *b* = 0 for all sites, when the level of selection bias was high, the bias in ${\hat{\beta }}_{1}$ was −0.002 (in logit prevalence) for complete-case analysis with the mixed-effects model, as opposed to −0.173, -1.833, and −0.411 for MI with Models 1, 2, and 3 respectively. Similarly for ${\hat{\beta }}_{3}$ the resulted bias was 0.001 for complete-case analysis with the mixed-effects model, 0.056 for MI with Model 1, 1.203 for MI with Model 2, and 0.299 for MI with Model 3 (see Tables 2, 3, 4 and 5). The bias in ${\hat{\beta }}_{1}$ indicated a tendency to overestimate the magnitude of decline in HIV prevalence. On the other hand, the bias in ${\hat{\beta }}_{3}$ indicated the tendency to overestimate the difference in HIV prevalence trends between intervention and nonintervention sites.

**Table 2:** **Bias in**${\hat{\beta }}_{1}=E\left({\hat{\beta }}_{1}\right)-\beta_{1}$**,**$\beta_{1}=0$

**Level of selection bias**	**Complete-case (Fixed-effects)**	**Complete-case (Mixed-effects)**	**Model 1**	**Model 2**	**Model 3**
High	−0.080	−0.002	−0.173	−1.883	−0.411
Moderate	−0.001	0.002	−0.043	−0.887	−0.186
Low	−0.001	−0.001	−0.048	−0.715	−0.162

**Table 3:** **Bias in**${\hat{\beta }}_{3}=E\left(\hat{\beta_{1}}\right)-\beta_{1}$**,**$\beta_{3}=0$

**Level of selection bias**	**Complete-case (Fixed-effects)**	**Complete-case (Mixed-effects)**	**Model 1**	**Model 2**	**Model 3**
High	−0.021	0.001	0.056	1.203	0.299
Moderate	−0.019	−0.001	0.013	0.415	0.191
Low	−0.008	−0.0001	0.020	0.292	0.179

**Table 4:** **Probability coverage of 95% confidence intervals for**$\beta_{1}$**, **$\beta_{1}=0$

**Level of selection bias**	**Complete-case (Fixed-effects)**	**Complete-case (Mixed-effects)**	**Model 1**	**Model 2**	**Model 3**
High	0.038	0.884	0.059	0.035	0
Moderate	0.129	0.933	0.593	0.179	0.007
Low	0.12	0.924	0.603	0.685	0.032

**Table 5:** **Probability coverage of 95% confidence intervals for**$\beta_{3}$**, **$\beta_{3}=0$

**Level of selection bias**	**Complete-case (Fixed-effects)**	**Complete-case (Mixed-effects)**	**Model 1**	**Model 2**	**Model 3**
High	0.213	0.942	0.616	0.197	0
Moderate	0.114	0.952	0.766	0.566	0.007
Low	0.173	0.949	0.786	0.205	0.019

The probability coverage of 95% confidence intervals yielded by complete-case analysis with the mixed-effects model was the closest to the nominal level across all scenarios. The coverage for *β*_1_ and *β*_3_ ranged from 0.884 to 0.952. In contrast, the probability coverage yielded by various MI models was consistently below nominal level, ranging from 0 to 0.786. The poor probability coverage implies an increased risk of declaring a statistically significant trend or program impact when the effects are not present.

As demonstrated in the results, MI models were sensitive to the level of selection bias. As the severity of selection bias increased, the magnitude of bias increased and the probability coverage of the parameter decreased. The impact of selection bias was less pronounced in the complete-case analysis with the mixed-effects model; however, it could still be noted in the reduced probability coverage for *β*_1_ in the high selection bias scenario.

Complete-case analysis with the fixed-effects model did not yield satisfactory outcomes. Although the bias in parameter estimates was low relative to that of MI models, the probability coverage for the parameters was poor. When the level of selection bias was low, the probability coverage of 95% confidence intervals for *β*_1_ and *β*_3_ was below 0.12 and 0.173 respectively.

We focus on the high selection bias situation and examine how well the methods perform when temporal trends exist and differ between intervention and nonintervention sites. As shown in Table 6, complete-case analysis with a mixed-effects model continued to outperform other methods and yield the most unbiased coefficients estimates. The bias in ${\hat{\beta }}_{1}$ and ${\hat{\beta }}_{3}$ ranged from −0.024 to 0.001. In contrast, the bias resulted from the various MI models were substantially higher, ranging from −0.438 and −0.066. Overall, MI models tended to estimate declining trends, which were more dramatic than the actual ones. In terms of probability coverage, when *β*_1_ = 0 and *β*_3_ = –0.025, complete-case analysis with a mixed-effects model yielded the best results. The coverage of the confidence intervals for the coefficients was 0.924 and 0.833, respectively (see Table 7). The coverage was less satisfactory when *β*_1_ = –0.025 and *β*_3_ = –0.025. In that scenario, complete-case analysis with fixed-effects and two of the MI models yielded better coverage. However, these results must be interpreted with care. As the coefficient estimates yielded by these methods were severely biased, the probability coverage has limited implication on the performance of the methods.

**Table 6:** **Bias in**${\hat{\beta }}_{1}$**and**${\hat{\beta }}_{3}$**for scenarios with differential trends in intervention and non-intervention sites (with a high level of selection bias)**

**True parameters**	**Complete-case (Fixed-effects)**	**Complete-case (Mixed-effects)**	**Model 1**	**Model 2**	**Model 3**
Scenario 1					
*β*_1_ = 0	−0.082	−0.002	−0.182	−1.983	−0.438
*β*_3_ = –0.025	−0.037	−0.024	−0.092	−1.388	−0.362
Scenario 2					
*β*_1_ = –0.025	−0.085	−0.002	−0.181	−1.764	−0.415
*β*_3_ = –0.025	−0.006	0.001	−0.066	−1.188	−0.307

**Table 7:** **Probability coverage of 95% confidence intervals for**$\beta_{1}$**and**$\beta_{3}$**for scenarios with differential trends in intervention and non-intervention sites (with a high level of selection bias)**

**True parameters**	**Complete-case (Fixed-effects)**	**Complete-case (Mixed-effects)**	**Model 1**	**Model 2**	**Model 3**
Scenario 1					
*β*_1_ = 0	0.183	0.924	0.049	0.039	0.715
*β*_3_ = –0.025	0.839	0.833	0.657	0.941	0.069
Scenario 2					
*β*_1_ = –0.025	0.854	0.462	0.897	0.969	0.325
*β*_3_ = –0.025	0.757	0.349	0.541	0.897	0.088

### ANC HIV prevalence trends and the impact of the prevention program

Based on the ANC sentinel data obtained from NACO between 2002 and 2008, we examined the changes in HIV prevalence among ANC attendees and the potential impact of the prevention program. The results are shown in Table 8. Of particular interest are the coefficients ${\hat{\beta }}_{1}$ and ${\hat{\beta }}_{3}$ in the analysis model. These coefficients represent the changes in ANC HIV prevalence over time and the potential differences in the changes between intervention and nonintervention sites.

**Table 8:** **Analysis of changes in ANC HIV prevalence trend and the impact of program intervention using various imputation approaches and analysis model:***logit*(***p***_*st*_) = *β*_0_ + *β*_1_*t* + *β*_2_*I* + *β*_3_*I* × *t* + *α*_0*s*_ + *α*_1*s*_*t*

**Methods**	${\hat{\beta }}_{0}$	${\hat{\beta }}_{1}$	${\hat{\beta }}_{2}$	${\hat{\beta }}_{3}$
Complete-case (mixed-effects)	165.09 (117.21, 212.98)	−0.085 (−0.11, -0.06)	56.21 (−4.43, 116.86)	−0.027 (−0.058, 0.003)
Model 1	176.10 (136.91, 215.29)	−0.091 (−0.13, -0.05)	46.95 (−3.66, 97.55)	−0.023 (−1.16, 0.03)
Model 2	448.23 (−1932.11, 2828.57)	−0.229 (−2.77, 2.31)	550.47 (−3602.30, 4703.24)	−0.274 (−4.75, 4.20)
Model 3	402.2 (358.89, 445.42)	−0.203 (−0.25, -0.16)	−148.32 (−206.24, -90.40)	0.074 (0.01, 0.14)

1. National Aids Control Organisation and Government of India Ministry of Health Family Welfare: **ANNUAL REPORT 2008–2009**. In*.* New Delhi; 2009.

The ${\hat{\beta }}_{3}$ estimated by complete-case analysis with the mixed-effects model was −0.027 (CI: -0.058, 0.003), which was similar to that estimated by MI with Model 1, -0.023 (CI: -1.16, 0.03). Model 2 also yielded a negative ${\hat{\beta }}_{3}$ but with slightly larger magnitude: -0.274 (CI: -4.75, 4.20). None of the estimates was statistically significant. On the other hand, ${\hat{\beta }}_{3}$ estimated by Model 3 was positive and statistically significant (${\hat{\beta }}_{3}$ = 0.074, CI: 0.01, 0.14). This implies that the decline in ANC prevalence was more pronounced in nonintervention sites than in intervention sites.

With regard to the estimates of ${\hat{\beta }}_{1}$, all models indicated a negative HIV prevalence trend. The estimated ${\hat{\beta }}_{1}$ was −0.085 (CI: -0.107, -0.061) for complete-case analysis with the mixed-effects model, -0.091 (CI: -0.13, -0.05) for MI with Model 1, -0.229 (CI: -2.77, 2.31) for Model 2, and −0.203 (CI: -0.25, -0.16) for Model 3. The results from the complete-case analysis and MI with Model 1 suggested a statistically significant overall decline in ANC HIV prevalence over time. As for the results from Model 3, since ${\hat{\beta }}_{3}$ yielded by Model 3 was statistically significant, ${\hat{\beta }}_{1}$ reflected the changes in ANC HIV prevalence amongst the nonintervention sites. The result indicated that there was a significant decline in HIV prevalence in nonintervention sites.

We should emphasize that several studies have been carried out to evaluate the effectiveness of the program [21,22]. The goal here is merely to demonstrate the application of various approaches and not to offer a comprehensive evaluation of the program.

## Discussion

Selection bias as a result of the phasing in of clinics in national clinic-based HIV surveillance systems is a major problem. Despite this, methods for addressing the issue have not been well studied. In this paper, we demonstrated the impact of selection bias on analyses of HIV prevalence trends and intervention evaluation. Through a set of simulations, we compared the performances of complete-case analysis and multiple imputations with different model specifications.

Considering the extent of missing data, one might have expected that the application of multiple imputation would enhance the accuracy of the analysis. However, in our simulation study, the performance of MI was not completely satisfactory. There was considerable bias in the parameter estimates, and the confidence intervals derived did not offer the desirable probability coverage. One explanation for the unsatisfactory performance of MI may be that the assumption of ignorable missingness was not fulfilled. According to the data generation procedure in our simulation, the more severe the selection bias was, the closer the missing data pattern tended towards nonignorable missingness. As described in the results, the performance of the various MI models deteriorated as the level of selection bias increased. In other words, the MI models considered here are not resistant to the violation of the ignorable missingness assumption.

Our results also indicated that the choice of imputation models is critical. We compared three imputation models: Model 1 was a relatively general fixed-effects model; Model 2 was a mixed-effects model identical to the analysis model; and Model 3 was designed to take advantage of the monotone missing data pattern. Substantial discrepancies existed in the performance of the three models with Model 1 yielding relatively better outcomes. Nevertheless, the performance of all the models was below optimal. Rubin [19]pointed out the statistical issues associated with imputing data with nonignorable missing values. In situations where the nonresponse mechanism is not properly taken into account, imputation of missing data may fail. Several recent studies have indicated that caution should be exercised when using MI in epidemiological and clinical studies [23,24].

For the present situation, the complete-case analysis with mixed-effects model performs the best. It has often been suggested that complete-case analysis can yield biased estimates and loss of efficiency [25,26]. However, the findings here suggest that as long as an appropriate analysis model is adopted, complete-case analysis can yield unbiased parameter estimates and desirable probability coverage.

We applied the various methods to analyze the changes in HIV prevalence among ANC attendees in India. The variation in the outcomes yielded by the different methods was remarkable. Taking into consideration findings from the simulation, the estimates based on complete-case analysis with a mixed-effects model may be more reliable. The method found no significant difference in the changes of ANC HIV prevalence between sites with and without intervention. However, a significant decline in the overall ANC HIV prevalence trend was detected. Similar results were obtained by MI with Model 1. The similarities in the findings of the complete-case analysis and Model 1 suggested that the impact of selection bias in this case is perhaps rather mild.

Despite the potential biases in clinic-based surveillance data, such data are often the only source available for continuous HIV prevalence time series and continue to be an important tool for monitoring HIV prevalence around the world [11]. In recent years, many countries have reported a drastic decline in HIV prevalence based on these surveillance programs, and some studies have associated the decline with successful implementation of HIV prevention programs. For example, in Kenya, data from sentinel surveillance indicated a rapid decline in national prevalence from 7.5% in 2001 to 6.7% in 2003 [13]. In a study in Addis Ababa, Ethiopia, it was reported that the prevalence of HIV infection among women attending ANC declined from an average of 21.2% to 15.6% from 1995 to 2001 [27]. A study in Cameroon [28] suggested that the HIV prevalence of antenatal clinic attendees in two provinces decreased significantly from 11% in 2000 to about 8% in 2006 (p < 0.001). In India, a study showed that HIV prevalence rates among female sex workers were reduced by nearly half in four years [29]. In a similar study, the HIV prevalence among ANC attendees was found to decline from 1.4% to 0.77%. In intervention-intensive locations, the percent decline was as high as 56% compared to only 5% in nonintervention locations [21]. In these studies, the rationale behind the choice of analysis methods was not always explicit, and the adequacy of those methods in addressing the issue of selection bias was not immediately apparent. Given how sensitive methods can be toward selection bias, it is unclear to what extent the decline in HIV prevalence estimated was a result of the intervention implemented and to what extent it was the effect of selection bias.

In conclusion, caution must be taken when analyzing data from clinic-based surveillance systems. Failure to take selection bias into account can lead to biased estimation of the magnitude of declines in HIV prevalence and the impact of an intervention program. We have demonstrated that methods and models vary in their capacity to tackle selection bias. Data imputation procedures may not always be effective. Instead, if an appropriate analysis model is applied, complete-case analysis can be superior. From a practical standpoint, when determining the appropriate analysis strategy, it is recommended that researchers always cross-validate competing methods in order to better understand how the performance of a method may be affected by certain features in a dataset.

## Competing interests

The authors declare that they have no competing interests.

## Authors’ contributions

MN developed the method and drafted the paper. EG, CJLM, and SSL guided the method development and edited the paper. All authors read and approved the final manuscript.
